# Functional Analysis of the Collagen Binding Proteins of Streptococcus parasanguinis FW213

**DOI:** 10.1128/mSphere.00863-20

**Published:** 2020-10-14

**Authors:** Yi-Ywan M. Chen, Pei-Hua Tsai, Zong-Sian Ye, Yu-Wen Huang, Hui-Ru Shieh, Chia-Hua Wu, Yu-Juan Lin, James H. Miller, Jacqueline Abranches, Cheng-Hsun Chiu

**Affiliations:** a Department of Microbiology and Immunology, College of Medicine, Chang Gung University, Taoyuan, Taiwan; b Graduate Institute of Biomedical Sciences, College of Medicine, Chang Gung University, Taoyuan, Taiwan; c Molecular Infectious Disease Research Center, Chang Gung Memorial Hospital, Linkou, Taiwan; d Department of Biology, University of Rochester, Rochester, New York, USA; e Department of Oral Biology, University of Florida College of Dentistry, Gainesville, Florida, USA; University of Kentucky

**Keywords:** collagen binding proteins, *Streptococcus parasanguinis*, biofilm, phagocytosis, *Galleria mellonella* larva model

## Abstract

Streptococcus parasanguinis is a dominant isolate of dental plaque and an opportunistic pathogen associated with subacute endocarditis. As the expression of collagen binding proteins (CBPs) could promote the establishment of S. parasanguinis in the host, the functions of three putative CBP-encoding loci, Spaf_0420, Spaf_1570, and Spaf_1573, were analyzed using isogenic mutant strains. It was revealed that *S. parasanguinis* FW213 bound effectively to fibronectin and type I collagen, but the strain’s affinity for laminin and type IV collagen was quite low. By using various deletion derivatives, it was found that these three loci mediated the binding of *S. parasanguinis* to multiple extracellular matrix molecules, with type I collagen as the common substrate. Derivative strains with a deletion in any of the three loci expressed reduced binding to trypsin-treated swine heart valves. The deletion of these loci also reduced the viable count of *S. parasanguinis* bacteria within macrophages, especially the loss of Spaf_0420, but only strains with deletions in Spaf_0420 and Spaf_1570 expressed reduced virulence in the Galleria mellonella larva model. The deletion of Spaf_1570 and Spaf_1573 affected mainly the structure, but not the overall mass, of biofilm cultures in a flow cell system. Thus, CBPs are likely to be more critical for the initial colonization of *S. parasanguinis* on host tissues during the development of endocarditis.

**IMPORTANCE** Bacteria generally can utilize multiple adhesins to establish themselves in the host. We found that Streptococcus parasanguinis, a dominant oral commensal and an opportunistic pathogen for subacute endocarditis, possesses at least three collagen-binding proteins that enable *S. parasanguinis* to successfully colonize damaged heart tissues and escape innate immune clearance. The binding specificities of these three proteins for extracellular matrix molecules differ, although all three proteins participate in biofilm formation by *S. parasanguinis*. The “multiligand for multisubstrate” feature of these adhesins may explain the high adaptability of this microbe to different tissue sites.

## Abstract (precis)

Bacteria generally can utilize multiple adhesins to establish themselves in the host. We found that Streptococcus parasanguinis, a dominant oral commensal and an opportunistic pathogen for subacute endocarditis, possesses at least three collagen-binding proteins that enable *S. parasanguinis* to successfully colonize damaged heart tissues and escape innate immune clearance. The binding specificities of these three proteins for extracellular matrix molecules differ, although all three proteins participate in biofilm formation by *S. parasanguinis*. The “multiligand for multisubstrate” feature of these adhesins may explain the high adaptability of this microbe to different tissue sites.

## INTRODUCTION

The extracellular matrix (ECM) of mammals comprises approximately 300 proteins, including collagen proteins, proteoglycans, and complex glycoproteins (1). The ECM participates in multiple biological processes in eukaryotes (2) and is also the target for bacterial colonization (3), the key step for establishing an infection. Among mammalian ECM molecules, collagen proteins are the most abundant type and are present in all connective tissues (4). Pathogens can establish themselves on collagenous tissues through the activity of collagen binding proteins (CBPs) (5). CBPs in Gram-positive bacteria belong to a group of proteins with a common structure known as MSCRAMMs (microbial surface components recognizing adhesive matrix molecules) (6, 7). The general structure of MSCRAMMs includes an N-terminal leader peptide followed by a ligand binding domain (also known as the A region), a variable number of B domain repeats, and a cell wall sorting region (8). Although the function of B domain repeats is not fully defined, it has been suggested that sequences of the B repeats form isopeptides and, thus, can provide structural stability to the protein (7). Some CBPs bind collagen proteins only, whereas others may recognize multiple substrates. For example, both Cna of Staphylococcus aureus (9) and Ace of Enterococcus faecalis (10) bind only to collagen, and although Cna and Ace share a high degree of sequence similarity, these two proteins show different affinities for type I collagen (11–13). On the other hand, CbpA of Trueperella pyogenes (previously classified as Arcanobacterium pyogenes) binds collagen and fibronectin at different subsites in CbpA (14).

CBPs play important roles in the pathogenesis of streptococci (15). For instance, CpA of Streptococcus pyogenes binds to type I collagen, and CpA mutant strains exhibit decreased attachment to HEp-2 cells (16). In Streptococcus mutans, Cnm is required for adherence to and invasion of human coronary artery endothelial cells (17) and is associated with hemorrhagic stroke, cerebral microbleeds, and IgA nephropathy (18–20), whereas Cbm is associated with infective endocarditis (IE) (21). Other examples include CbpA of Streptococcus gordonii that can enhance the survival of S. gordonii *in vivo* (22). Furthermore, Cbp40 of Streptococcus suis serotype 2 is required for optimal adhesion to HEp-2 cells, biofilm formation, and virulence in the zebra fish infection model (23).

Streptococcus parasanguinis is an early colonizer and a common isolate from dental plaque (24). When S. parasanguinis gains access to the bloodstream, it can successfully colonize heart tissue and, thus, is a common cause of bacterial subacute endocarditis (25). Despite the significance of *S. parasanguinis* in the oral ecosystem and systemic infections, the known virulence factors of this microbe are limited to Fap1, BapA1, and FimA of the FimCBA ATP binding cassette transport system for Mn^2+^ and Fe^2+^. Specifically, both Fap1 (26), the structural subunit of the long fimbriae, and Bap1 (27), a surface protein, are associated with biofilm formation, whereas FimA participates in the development of IE in a rat model, presumably by binding to the fibrin monolayer (28, 29). Whether other MSCRAMMs also contribute to the development of IE by *S. parasanguinis* is yet to be determined. Here, we elucidate the functions of the putative CBPs in the colonization, biofilm formation, and survival of *S. parasanguinis* against macrophage clearance.

## RESULTS

### Identification and basic characterization of loci encoding CBPs.

**FIG 1:**
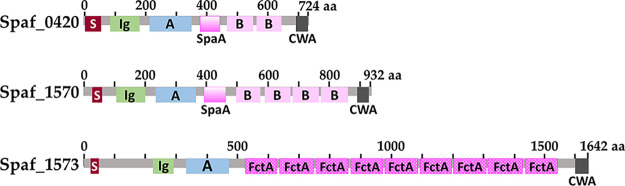
Schematic representation of the conserved domains in the CBP homologues of *S. parasanguinis* FW213. S, signal peptide; Ig, bacterial SDR-like Ig domain; A, collagen binding A domain; B, collagen binding B domain; CWA, LPXTG cell wall anchoring motif; SpaA, prealbumin-like fold domain; FctA, Spy0128-like isopeptide-containing domain.

**FIG 2:**
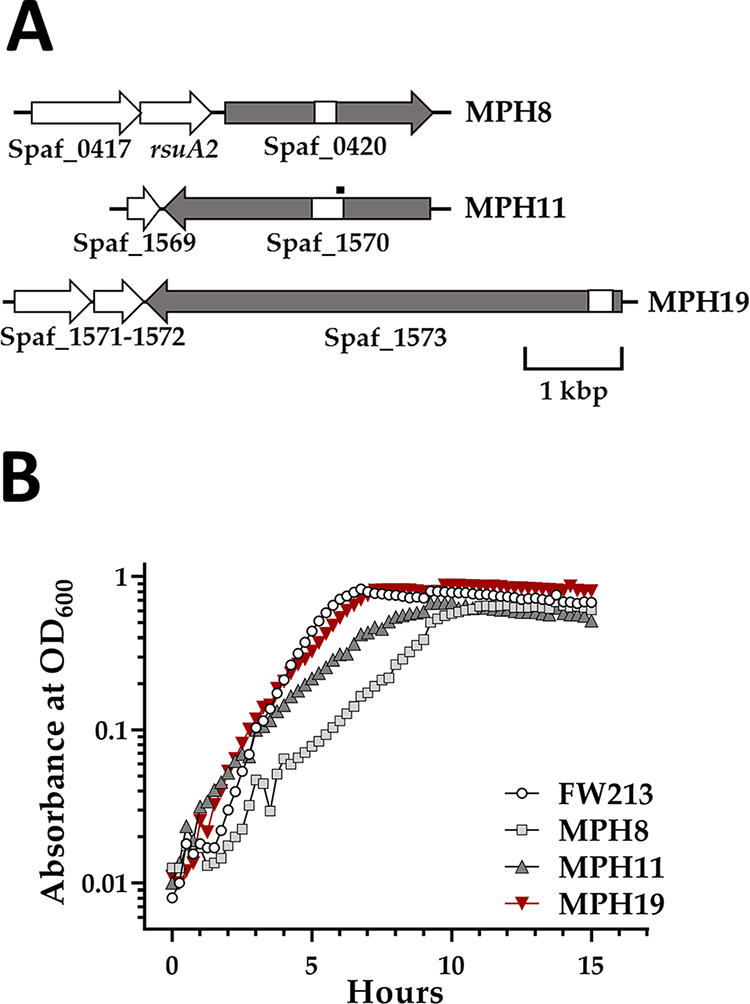
Genotype (A) and growth kinetics (B) of *S. parasanguinis* FW213 and its CBP-deficient derivatives. (A) Genes encoding the putative CBPs (in gray) and the flanking loci (in white). The gene name or the Spaf_tag number is listed below the gene. All loci are illustrated based on the orientation on the chromosome. The region that was replaced with *erm* in MPH8, MPH11, and MPH19 is indicated by a white box. The region in Spaf_1570 that was replaced with Ω*kan* in ZC1 and ZC2 is indicated by a thick horizontal line above the gene. (B) Growth of *S. parasanguinis* strains in TH broth. A graph representative of data from three experiments is shown.

Three putative CBP-encoding loci, Spaf_0420, Spaf_1570, and Spaf_1573, were identified from the genome of *S. parasanguinis* FW213 (30) by a domain search and BLAST using sequences from known CBPs. The basic characteristics of these open reading frames (ORFs) are listed in Table 1. *In silico* sequence analyses indicated that all three proteins harbor a multidomain architecture similar to those of the known MSCRAMMs (Fig. 1). In addition to the collagen binding A domain and the B domain repeats, all three ORFs harbor an SDR (serine-aspartate repeat-containing protein family)-like immunoglobulin (Ig) domain that is frequently found in bacterial surface adherence proteins, including the Ace collagen binding protein of E. faecalis (12). Furthermore, the SpaA domain, named after the shaft pilin SpaA of Corynebacterium diphtheriae (31), and the FctA domain, known as the FCT (fibronectin-collagen-T antigen) pilus locus of S. pyogenes (32), were observed. Both domains are also found on a variety of bacterial surface proteins, including ECM binding proteins. Similar to the B domain repeats, structures of both SpaA and FctA domains contain intramolecular isopeptides (31, 33). All three genes were insertionally inactivated to generate single-, double-, or triple-knockout strains (Fig. 2A), as detailed in Materials and Methods, to define their functions.

**TABLE 1:** Properties of the putative CBPs in *S. parasanguinis* FW213

Locus	Length (nt)	RBS sequence (distance [nt])^*a*^	Protein size (aa/kDa)	pI	Identity (aa/total aa; % identity)^*b*^	GenBank accession no.^*c*^
Spaf_0420	2,175	GAAAAAAG (7)	724/79.56	5.47	Cna B-type domain-containing protein in Streptococcus australis (554/694; 80)	WP_006595581.1
Spaf_1570	2,799	GGAAAGGG (7)	932/104.29	5.51	Cna B-type domain-containing protein in Streptococcus australis (499/889; 56)	WP_006595427.1
Spaf_1573	4,929	GAAAG (6)	1,642/177.75	4.95	YSIRK signal domain/LPXTG anchor domain surface protein in Streptococcus infantis (1,325/1,635; 81)	WP_045763103.1

a The distance between the ribosome binding site (RBS) and the translational start site is listed in parentheses. nt, nucleotides.

b The target to which the ORF shares the highest homology. The length of the alignment over the target size and the percent identity at the deduced amino acid level are listed in parentheses.

c GenBank accession number of the locus listed in the identity column.

To determine whether mutations in the putative CBPs would affect the growth of *S. parasanguinis*, the growth kinetics of wild-type FW213 and mutant strains carrying single knockouts were examined (Fig. 2B). Although all strains reached similar final optical density at 600 nm (OD_600_) values, the inactivation of any of the three ORFs reduced the growth rate of *S. parasanguinis*. The estimated generation time of wild-type FW213 was 57 ± 5 min, whereas the doubling times of MPH8, MPH11, and MPH19 were 92 ± 8, 82 ± 6, and 80 ± 6 min, respectively.

### Adherence of *S. parasanguinis* FW213 to ECM proteins.

**FIG 3:**
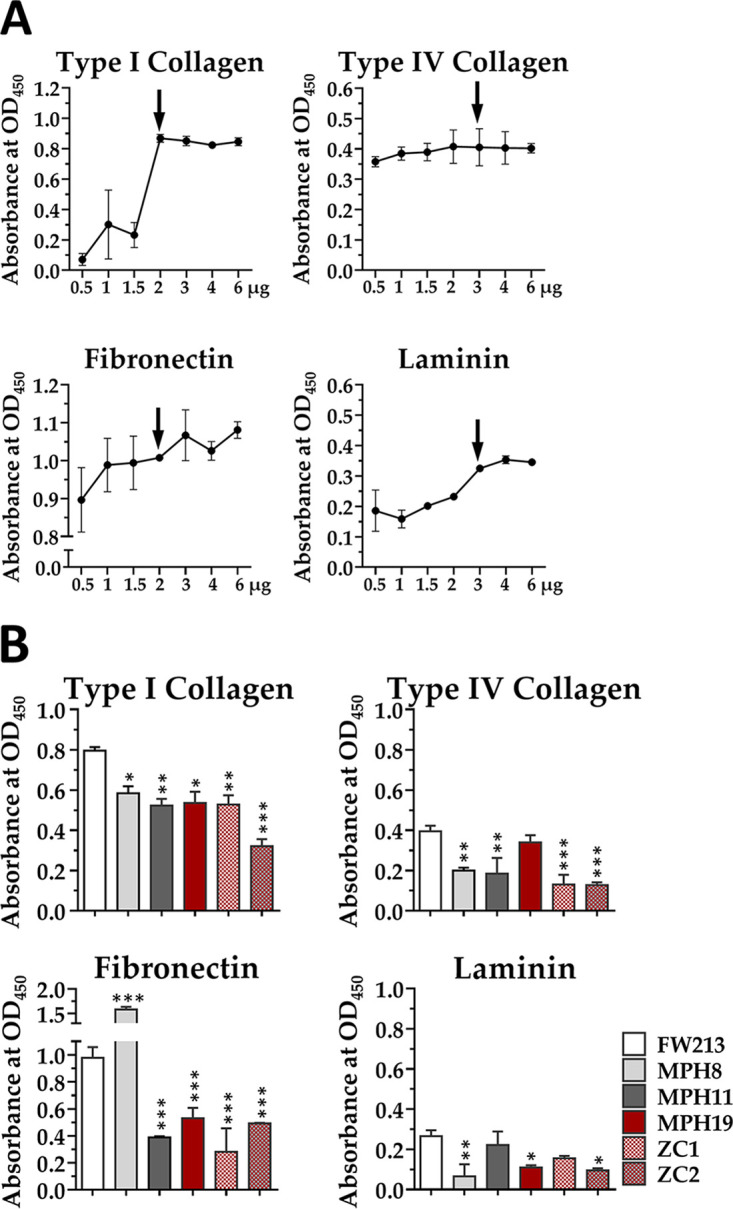
ELISA demonstrating the binding of *S. parasanguinis* FW213 to ECM molecules. (A) Dose-dependent adherence of *S. parasanguinis* FW213 to ECM molecules. Wells of the ELISA plate were coated with different amounts of ECM molecules. The bound bacteria were detected by a monoclonal antibody specific for *S. parasanguinis* FW213. The amount of the ECM molecule used to analyze the binding efficiency in panel B is indicated by vertical arrows. Wells without the addition of bacteria were used as a background control. The values are the means and standard deviations from three independent experiments. (B) Adherence of *S. parasanguinis* FW213, MPH8, MPH11, MPH19, ZC1, and ZC2 to ECM molecules. The values are the means and standard deviations from three independent experiments. Significant differences between wild-type FW213 and its derivatives were determined by ANOVA followed by Dunnett’s test. ***, *P < *0.0001; **, *P < *0.001; *, *P < *0.01.

A binding curve for type I collagen, type IV collagen, fibronectin, and laminin was initially determined in wild-type *S. parasanguinis* FW213 by an enzyme-linked immunosorbent assay (ELISA). Maximal binding was observed when wells were coated with 2 to 3 μg of the ECM substrates (Fig. 3A). The results also indicated that *S. parasanguinis* FW213 bound to type I collagen and fibronectin most effectively, with a relatively low affinity for type IV collagen and laminin.

The inactivation of any of the three ORFs (MPH8, MPH11, and MPH19 strains) reduced the binding of *S. parasanguinis* to type I collagen (Fig. 3B). Although the ZC1 strain (where both Spaf_1570 and Spaf_1573 are inactivated) exhibited a binding level comparable to those of strains carrying a single knockout, the inactivation of all three ORFs (strain ZC2) resulted in the lowest binding level observed. The difference in the binding levels between ZC2 and the other mutant strains was further confirmed by analysis of variance (ANOVA) followed by Tukey’s test (*P < *0.01). Thus, it is suggested that all three ORFs participate in binding to type I collagen and that Spaf_0420 may interact with the substrate at a site different from those of the other two ORFs. With the exception of MPH19 (with Spaf_1573 inactivated), all mutant strains showed reduced binding to type IV collagen. All mutant strains, except MPH8 (with Spaf_0420 inactivated), exhibited a significant reduction in binding to fibronectin compared to wild-type FW213. Unexpectedly, enhanced binding to fibronectin was observed in MPH8 (Fig. 3B); the nature of this phenotype is currently unknown. On the other hand, MPH8, MPH19, ZC1, and ZC2 exhibited reduced binding to laminin compared to wild-type FW213, indicating that Spaf_0420 and Spaf_1573 mediated binding to laminin. Finally, as ZC2 remained able to bind to type I collagen and fibronectin, *S. parasanguinis* FW213 is likely to possess additional ligands for these substrates. Taken together, the data demonstrate that Spaf_0420 participates in binding to type I collagen, type IV collagen, and laminin; Spaf_1570 participates in binding to type I and type IV collagen and fibronectin; and Spaf_1573 participates in binding to type I collagen, fibronectin, and laminin. Thus, all three ORFs were able to bind to multiple substrates, with type I collagen being the common substrate for all CBPs tested.

### Role of CBP homologues in *S. parasanguinis* binding to damaged swine heart tissues.

**FIG 4:**
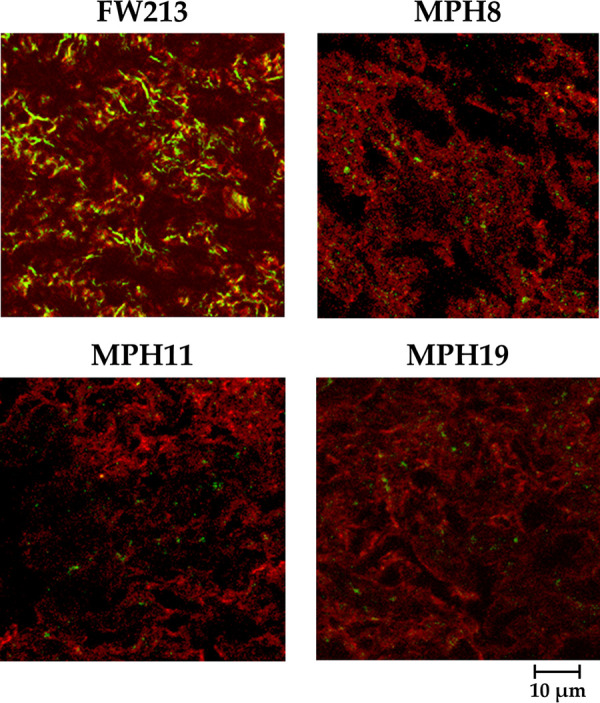
Examination of the binding of *S. parasanguinis* FW213 and its derivatives to swine heart tissues by CLSM. Type I collagen fibers on trypsin-treated swine heart tissues are shown by rhodamine-conjugated IgG (red), and the bacterial cells are labeled with FITC-conjugated IgG (green). A similar result was observed in at least 15 fields with all strains.

As type I collagen is the major component of normal human mitral valves (34), all three CBPs are likely to contribute to the colonization and development of endocarditis by *S. parasanguinis*. This hypothesis was tested using trypsin-treated swine heart valves. Confocal laser scanning microscopy (CLSM) examination indicated that all mutant strains bound less efficiently to the damaged valve tissues than wild-type FW213 (Fig. 4), confirming the activity of these proteins in the colonization of host tissues. All *S. parasanguinis* strains were recognized by the anti-lipoteichoic acid antibody at similar efficiencies using a dot blot assay. Additionally, we found that FW213 failed to bind to intact swine heart tissues (data not shown), indicating that exposure of ECM proteins through trypsin treatment is essential for colonization by *S. parasanguinis*.

### Impact of CBPs on biofilm formation of *S. parasanguinis* FW213.

**FIG 5:**
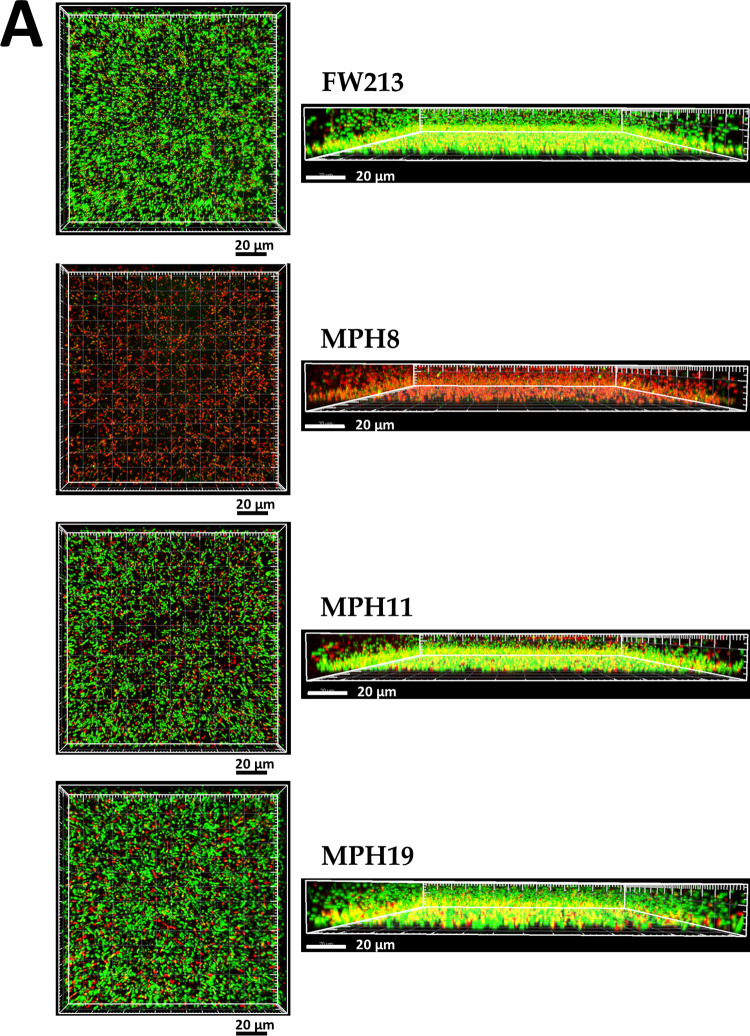

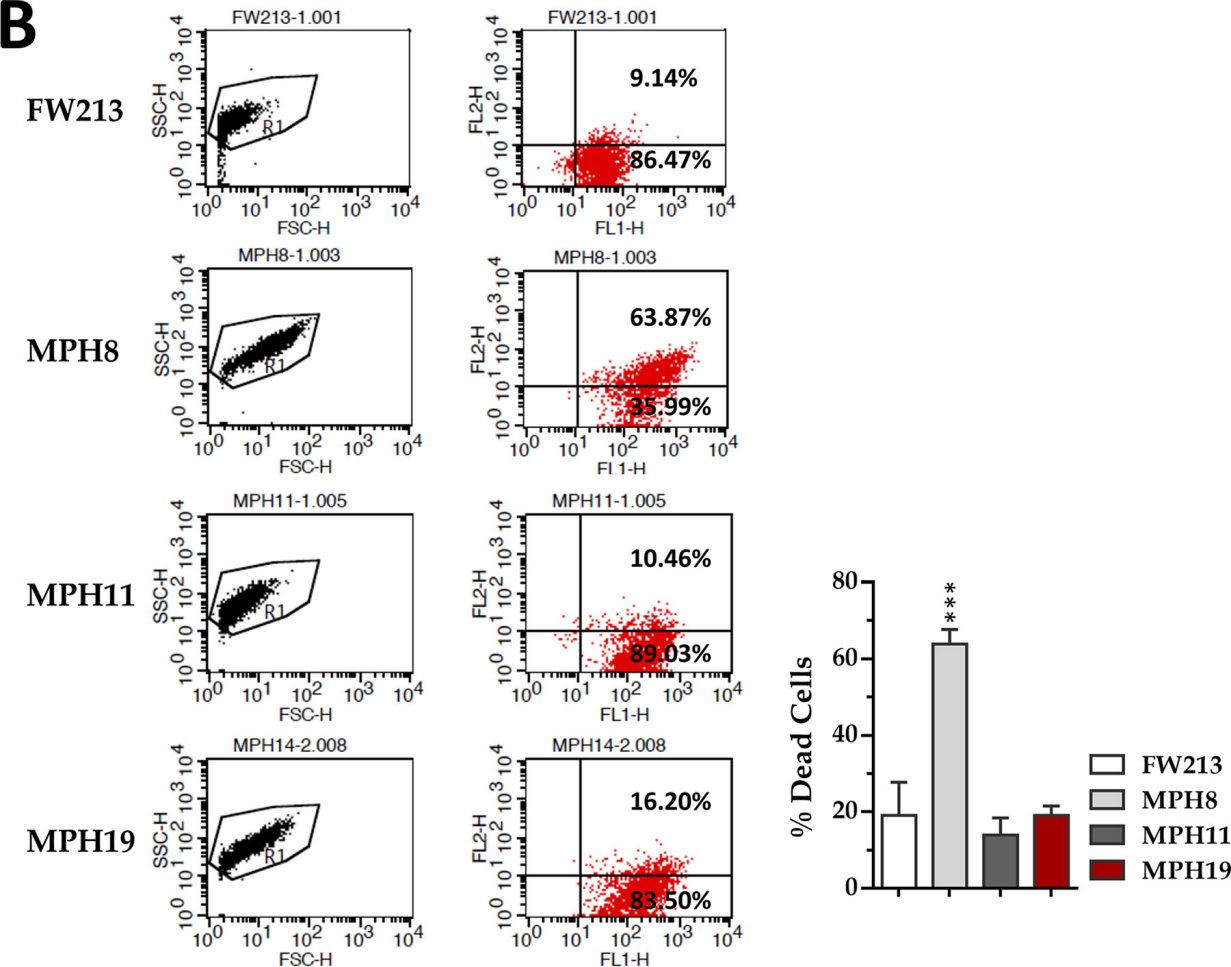
Role of Spaf_0420, Spaf_1570, and Spaf_1573 in static biofilm formation of *S. parasanguinis* FW213. (A) CLSM examination of the biofilm. The biofilms were stained with Live/Dead stain and examined at a ×64 magnification. The live and dead cells are indicated by green fluorescent (SYTO-9 stain) and red fluorescent (PI) staining, respectively. Top and side views are shown. (B) Flow cytometry analysis of the live and dead cells in the static biofilm. Approximately 10,000 cells were subjected to fluorescence analysis, and representative histograms are shown on the left. The values of percent gated cells of the righthand upper (dead cells) and lower (live cells) quadrants are indicated. SSC-H, side scatter-height; FSC-H, forward scatter-height; FL1-H, green fluorescence-height; FL2-H, red floursecence-height. The means and standard deviations for the upper right quadrant from three independent experiments are shown. Significant differences between wild-type FW213 and its derivatives were determined by ANOVA followed by Dunnett’s test. ***, *P < *0.0001.

To analyze whether the deletion of these CBP-encoding genes could affect the growth and structure of the biofilm, the distribution of live and dead cells in a biofilm culture was determined by staining and CLSM examination using both static and flow cell systems. In the static system, MPH8 formed a biofilm containing a majority of dead cells, compared to wild-type FW213 (Fig. 5A). Slightly larger amounts of dead cells were also observed in the biofilms of MPH11 and MPH19 (Fig. 5A). The number of dead cells in each of the biofilms was further analyzed by flow cytometry (Fig. 5B). In agreement with the observations from CLSM analyses, biofilm cultures of MPH8 contained the largest amounts of dead cells among the four strains, whereas the percentages of dead cells in the biofilms of MPH11 and MPH19 were similar to those found in wild-type FW213, indicating that Spaf_0420 was critical for the viability of *S. parasanguinis* FW213 in a static biofilm.

**FIG 6:**
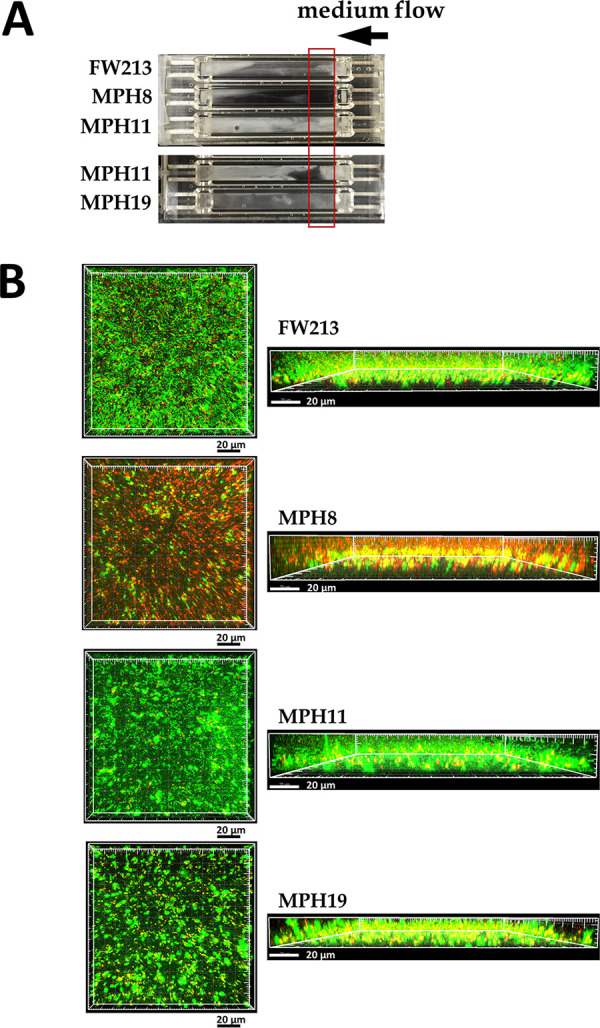
Biofilms of *S. parasanguinis* FW213 and its CBP-deficient derivatives in a flow cell system. (A) Gross examination of the biofilm in the flow chamber. The direction of medium flow is indicated by a horizontal arrow above the chamber. The area of each sample examined by CLSM in panel B is indicated by a red box. (B) CLSM examination of the biofilm. The biofilms were stained and examined as described in the legend of Fig. 5A.

A gross examination of the biofilm in a flow cell system revealed that biofilms created by MPH8 and MPH19 were thinner than those formed by wild-type FW213, whereas MPH11 was most capable of resisting the shearing force from the medium flow and formed the thickest biofilm among all strains (Fig. 6A). Similar to the static system, the biofilm of MPH8 was comprised mostly of dead cells (Fig. 6B). The views of the *x-y* plane of the biofilms created by the MPH11 and MPH19 strains suggested that the inactivation of Spaf_1570 or Spaf_1573 promoted cell-cell aggregation, which could result in a less structured biofilm. Taken together, the results suggest that all the CBPs were required for the fitness and subsequent biofilm formation of *S. parasanguinis*, especially the product of Spaf_0420.

### Inactivation of Spaf_0420 and Spaf_1570 results in reduced virulence of *S. parasanguinis* FW213 in the G. mellonella model.

**FIG 7:**
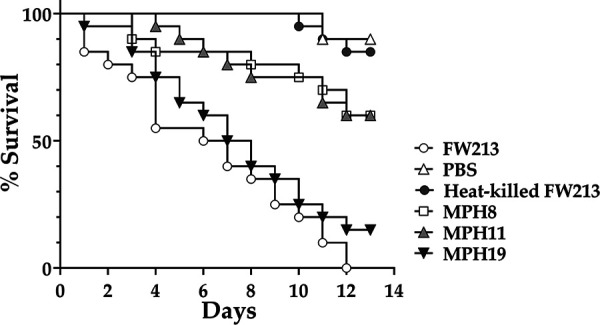
Virulence of *S. parasanguinis* strains in the G. mellonella larva model. A heat-killed *S. parasanguinis* FW213 cell suspension and PBS were used as background controls. Twenty larvae were used for each bacterial strain. Log rank survival analysis shows a significant reduction in virulence of *S. parasanguinis* strains MPH8 and MPH11 (*P < *0.0001). The data are representative of results from three independent experiments.

To analyze whether the CBPs contribute to the virulence of *S. parasanguinis* FW213, all strains were tested in the Galleria mellonella larva model of systemic infection (Fig. 7). The highest mortality rate was observed with larvae infected with the wild-type strain FW213. The mortality of larvae infected with MPH19 was similar to that of larvae infected with FW213, whereas >50% of the larvae (12 out of 20 larvae) remained viable after infection with MPH8 and MPH11, indicating that both Spaf_0420 and Spaf_1570 are important for the virulence of *S. parasanguinis* in systemic infections.

### CBPs are essential for survival of *S. parasanguinis* within macrophages but are not involved in macrophage recognition.

**FIG 8:**
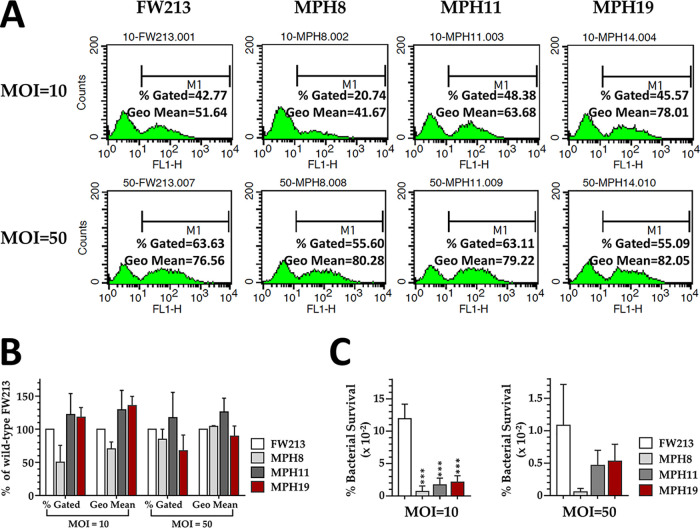
Impact of CBPs on the capacity of *S. parasanguinis* to clear immune clearance. (A) Flow cytometry analysis of the phagocytosis of *S. parasanguinis* FW213 and its derivatives by Raw264.7 macrophages. Representative histograms of Raw264.7 macrophages infected with *S. parasanguinis* are shown. Approximately 10,000 cells were subjected to fluorescence analysis. The percent gated population (percentage of the population with internalized bacteria) and the Geo mean (bacterial load per macrophage) of the M1 region for each analysis are shown. (B) The percent gated and Geo mean values obtained from the CBP deletion derivatives were normalized with the values from wild-type FW213. The means and standard deviations from three independent experiments are shown. (C) Raw264.7 macrophages were infected with *S. parasanguinis* strains at MOIs of 10 and 50. The survival rate was calculated as a percentage of the recovered bacterial count compared to the number of bacteria used in each infection. The values are the means and standard deviations from three independent experiments. Significant differences between wild-type FW213 and the mutant strains were determined by ANOVA followed by Dunnett’s test. ***, *P < *0.0001.

To ascertain whether the CBPs were recognized by macrophages during phagocytic clearance, the amount of internalized bacterial cells was quantified at 15 min postinfection by flow cytometry analysis (Fig. 8A). At a multiplicity of infection (MOI) of 10, the percentages of the gated population measured from macrophages infected with FW213, MPH11, and MPH19 were comparable (42 to 48%), whereas fewer (20%) macrophages had internalized MPH8 cells. At an MOI of 50, the percent gated population increased among all strains tested, with only minor differences between strains, presumably due to maximal loading of macrophages with bacteria. To analyze whether the differences observed between MPH8 and other strains at an MOI of 10 were significant, the relative internalization efficiency of each strain was calculated, compared to wild-type FW213 (set at 100%), from three independent experiments. Although the average of the percent gated population in MPH8 was lower than that of wild-type FW213, there was no significant difference between strains (Fig. 8B). We next determined the bacterial load per macrophage by examining the geometric mean (Geo mean). The trend of the Geo means between strains at each of the MOIs tested was the same as that seen in the percent gated population. Thus, Spaf_0420, Spaf_1570, and Spaf_1573 of *S. parasanguinis* FW213 were not the primary targets for macrophage recognition.

We next asked whether the CBPs also impact the survival of *S. parasanguinis* FW213 within macrophages. At an MOI of 10, the survival rates of all three CBP-deficient strains were significantly lower than that of wild-type FW213 (Fig. 8C). At an MOI of 50, the survival rates were approximately 10-fold lower than those observed at an MOI of 10 in all strains. Although the average survival rates of the mutant strains remained lower than that of wild-type FW213, the differences are not statistically significant (all *P* values are greater than 0.01). The lower bacterial survival rates at an MOI of 50 were likely the result of increased amounts of extracellular bacteria and toxicity from the bacteria compared to those at an MOI of 10. Taken together, the data show that the deletion of the CBP-encoding loci compromises the capacity of *S. parasanguinis* to survive within macrophages at a low MOI.

## DISCUSSION

*S. parasanguinis* FW213 is not unique in possessing multiple CBPs. For instance, 5 putative CBP-encoding loci (SSA_0227, SSA_0805, SSA_1019, SSA_1663, and SSA_1666) are present in the genome of Streptococcus sanguinis SK36, although the function of these loci has not been characterized. However, it was unexpected that all three CBPs analyzed in this study were specific for other ECM proteins besides collagen. Furthermore, we observed that the triple mutant strain (ZC2) possessed approximately 50% of the wild-type level of binding to all substrates tested, suggesting the presence of additional binding proteins. Presumably, the “multiligand for multisubstrate” feature could facilitate colonization by *S. parasanguinis* on different host tissues. As seen in S. aureus, the fibronectin binding proteins FnbpA and FnbpB recognize fibronectin, fibrinogen, and elastin, which can facilitate the establishment of an infection by the microbe in a variety of host species and tissue types (35). We were unable to identify additional CBPs in the *S. parasanguinis* FW213 genome based on sequence analysis thus far; however, a putative fibronectin binding protein, Spaf_1409, was found to share 88% identity with *fbpA* of S. sanguinis NCTC3168 at the deduced amino acid level. Our recent study shows that this locus is required for the optimal binding of *S. parasanguinis* FW213 to fibronectin (36), and thus, FW213 possesses at least four ECM binding proteins.

Our analyses on the binding of *S. parasanguinis* strains to host tissues (Fig. 4) and biofilm formation (Fig. 6) suggest that of the identified CBPs, at least Spaf_1570 and Spaf_1573 are more critical in the initial establishment of IE but not in the growth of the vegetation. Nevertheless, the compromised fitness in the CBP-inactivated mutant strains could also contribute to the reduced colonization of swine heart tissue. To analyze whether the deletion of the CBPs would reduce envelope integrity, we tested the resistance of the CBP deletion strains using an autolysis assay (37). We observed elevated lysis rates in the CBP deletion derivatives (data not shown). Since the N-terminal signal peptides remain intact in the mutant strains, it is possible that the truncated proteins in the recombinant mutant strains cause significant cell envelope stress between the cell membrane and cell wall and, subsequently, reduced viability. On the other hand, we did not observe significant binding of *S. parasanguinis* to intact swine heart tissues, confirming that a preexisting heart abnormality is a key factor for IE by oral streptococci.

Extracellular polymeric substances, such as extracellular DNA (eDNA), proteins, and polysaccharides, play important roles in the development and stability of biofilm (38). Thus, polymeric substances released from dead cells may potentially enhance biofilm formation. However, the addition of an excess amount of FW213 chromosomal DNA or DNase I in the biofilm culture did not significantly alter the final biofilm mass of wild-type FW213 (see Fig. S1 in the supplemental material), suggesting that eDNA only marginally impacts biofilm formation by *S. parasanguinis*. This is supported by the fact that larger amounts of eDNA and extracellular proteins were detected routinely in the biofilm cultures derived from MPH8 and MPH19 than in wild-type FW213, yet an elevated biofilm mass was detected only in MPH11 cultures (Fig. S2). We also found that, similar to wild-type FW213, neither excess amounts of eDNA nor DNase I treatment significantly affected the biofilm mass from MPH11 (Fig. S1). On the other hand, as the surface hydrophobicity of oral streptococci could affect their adherence to the salivary pellicle (39) and their interaction with other oral microbes (40), we thought that perhaps truncated Spaf_1570 in MPH11 had enough of an impact on surface hydrophobicity, leading to enhanced cell-cell interactions. As seen in a study by Nakao and colleagues, elevated surface hydrophobicity caused by truncated lipopolysaccharide (LPS) could enhance biofilm formation by Escherichia coli (41). When measuring the surface hydrophobicity of all strains used in this study using a xylene hydrophobicity assay (42), elevated hydrophobicity was observed only in MPH11 (Fig. S3). Thus, it is tempting to suggest that the truncation of Spaf_1570 in MPH11 was responsible for the elevated surface hydrophobicity and biofilm mass.

10.1128/mSphere.00863-20.1 FIG S1 Impact of extracellular DNA and DNase I treatment on biofilm formation of *S. parasanguinis* FW213 and MPH11. Static biofilm cultures were prepared as previously described (Y.-Y. M. Chen, Y.-Y. Chen, J.-L. Hung, P.-M. Chen, and J.-S. Chia, PLoS One 11:e0159599, 2016, https://doi.org/10.1371/journal.pone.0159599). Biofilms were grown in BMG without (control) or with the addition of 400 ng chromosomal DNA of FW213 or DNase I at 5 U per sample for 20 h. The mass of the biofilm was quantified according to the method of G. A. O’Toole and R. Kolter (Mol Microbiol 28:449–461, 1998, https://doi.org/10.1046/j.1365-2958.1998.00797.x). Values are the means and standard deviations from three independent experiments. Each assay was performed with triplicate samples. ns, not significant (*P > *0.05). Download FIG S1, TIF file, 0.5 MB. Copyright © 2020 Chen et al. 2020 Chen et al. This content is distributed under the terms of the Creative Commons Attribution 4.0 International license.

10.1128/mSphere.00863-20.2 FIG S2 Biofilm formation of *S. parasanguinis* FW213 and its CBP-deficient derivatives. (I) Static biofilms of bacteria grown in BMG were prepared and quantified as described in the legend of Fig. S1 in the supplemental material. (II) The amount of extracellular DNA (eDNA) in the biofilm cultures was estimated by quantitative real-time PCR using primers specific for the 16S rRNA genes of *S. parasanguinis* FW213. (III) Extracellular protein (eProtein) in the biofilm cultures was measured according to the method of M. M. Bradford [Anal Biochem 72:248–254, 1976, https://doi.org/10.1016/0003-2697(76)90527-3] using BMG as the background control. The values are the means and standard deviations from three independent experiments. Each assay was performed with triplicate samples. Significant differences between wild-type FW213 and its derivatives were analyzed using one-way ANOVA followed by Dunnett’s test. ***, *P < *0.0001; **, *P < *0.001; *, *P < *0.01. Download FIG S2, TIF file, 0.5 MB. Copyright © 2020 Chen et al. 2020 Chen et al. This content is distributed under the terms of the Creative Commons Attribution 4.0 International license.

10.1128/mSphere.00863-20.3 FIG S3 Surface hydrophobicity of *S. parasanguinis* FW213 and its CBP-deficient derivatives examined by a xylene assay [G. Mehta and B. Prakash, J Hosp Infect 20:129–130, 1992, https://doi.org/10.1016/0195-6701(92)90120-B]. Cultures grown overnight in BMG were used in the assay. The percentage of hydrophobicity was calculated as [1 − (OD_600_ after vortexing/OD_600_ before vortexing)] × 100. The values are the means and standard deviations from four independent experiments. Duplicate samples were used in each experiment. Significant differences between wild-type FW213 and its derivatives were analyzed using one-way ANOVA followed by Dunnett’s test. **, *P < *0.001. Download FIG S3, TIF file, 0.5 MB. Copyright © 2020 Chen et al. 2020 Chen et al. This content is distributed under the terms of the Creative Commons Attribution 4.0 International license.

Insects such as G. mellonella possess a complex, multicomponent innate immune system that is similar to the system used by mammals (43). Thus, the G. mellonella larva model has been widely used to study the virulence of a range of bacterial and fungal pathogens (44). As all three CBPs are not involved in the recognition of macrophages but could impact survival against clearance by macrophages, especially at lower MOIs (MOI = 10), the reduced virulence of MPH8 and MPH11 in larvae suggests that Spaf_0420 and Spaf_1570 are required for resistance to clearance by oxidative burst and other antimicrobial peptides in G. mellonella.

In conclusion, this study demonstrates that the CBPs of *S. parasanguinis* are essential for binding to the host ECM, the colonization of damaged heart tissues, and the evasion of host innate immune surveillance. The differential impacts of Spaf_0420, Spaf_1570, and Spaf_1573 on biofilm formation and larval killing may reflect distinct roles for these proteins in the oral cavity and the bloodstream.

## MATERIALS AND METHODS

### Bacterial strains and growth conditions.

All strains used in this study are listed in Table 2. *S. parasanguinis* strains were cultivated in Todd-Hewitt (TH) broth in the presence of 10% CO_2_ at 37°C. Erythromycin (Em) at 10 μg ml^−1^, kanamycin (Km) at 250 μg ml^−1^, and spectinomycin (Sp) at 500 μg ml^−1^ were used, where indicated, to maintain recombinant *S. parasanguinis* strains. To evaluate biofilm formation, strains were grown in biofilm medium (BM) (45) supplemented with 20 mM glucose (BMG).

**TABLE 2:** Bacterial strains used in this study

*S. parasanguinis* strain	Relevant phenotype	Description	Reference
FW213		Wild-type strain	56
MPH8	Em^r^; Spaf_0420 null	Spaf_0420::*erm* in FW213	This study
MPH11	Em^r^; Spaf_1570 null	Spaf_1570:: *erm* in FW213	This study
MPH19	Em^r^; Spaf_1573 null	Spaf_1573:: *erm* in FW213	This study
ZC1	Em^r^ Km^r^; Spaf_1570 and Spaf_1573 null	Spaf_1570::Ω*kan* in MPH19	This study
ZC2	Em^r^ Km^r^ Sp^r^; Spaf_0420, Spaf_1570, and Spaf_1573 null	Spaf_0420::*spe* in ZC1	This study

Recombinant E. coli strains were grown at 37°C with aeration in LB medium containing ampicillin (Ap) at 100 μg ml^−1^, Em at 200 μg ml^−1^, Km at 50 μg ml^−1^, Sp at 50 μg ml^−1^, or chloramphenicol (Cm) at 25 μg ml^−1^, as needed.

### *In silico* analysis of the putative CBP-encoding loci.

The putative CBP-encoding loci were extracted from the genome of *S. parasanguinis* FW213 (GenBank accession number CP003122.1). All ORFs were analyzed using Vector NTI advance 11 (Invitrogen), BLASTP suite (https://blast.ncbi.nlm.nih.gov/Blast.cgi), and Motif search (https://www.genome.jp/tools/motif/MOTIF3.html).

### Construction of isogenic mutant strains deficient in collagen binding homologues in *S. parasanguinis* FW213.

To generate recombinant strains with deletions in Spaf_0420, Spaf_1570, and Spaf_1573, sequences containing the coding regions or the 5′-flanking regions of the loci were generated from *S. parasanguinis* FW213 by PCR with specific primers. All fragments were cloned into plasmid pSU21 (46) and established in E. coli DH10B. The identities of the recombinant plasmids were verified by plasmid isolation and restriction endonuclease analysis. Inverse PCR was then performed with the correct recombinant plasmids to generate deletions within the loci and the concomitant introduction of restriction sites for the cloning of a polar Em resistance gene (*erm*) (47). The identity of the *erm-*containing plasmids was confirmed by sequence analysis, and the correct chimeric plasmids were introduced into *S. parasanguinis* FW213 by electroporation (48) to generate isogenic mutant strains via double-crossover recombination. The allelic-exchange event in the Em-resistant isolates was confirmed by colony PCR with primers outside the Em gene insertion site as well as sequencing of the interrupted loci. The resulting strains MPH8, MPH11, and MPH19 contain a deletion of amino acids (aa) 311 to 380 in Spaf_420, a deletion of aa 309 to 415 in Spaf_1570, and a deletion of aa 41 to 108 in Spaf_1573, respectively.

A similar approach was used to construct strains defective in both Spaf_1570 and Spaf_1573 and in all three loci. Briefly, the sequence encoding aa 308 to 328 of Spaf_1570 was replaced by a polar Km resistance gene (Ω*kan*) (49) in MPH19. The resulting strain, which bears deletions in both Spaf_1570 and Spaf_1573 and is resistant to both Km and Em, was designated ZC1. Amino acids 311 to 380 of Spaf_0420 were then replaced with a polar Sp resistance gene (*spe*) (50) in ZC1 to generate a triple-knockout mutant strain, ZC2.

### Growth kinetics.

The growth kinetics of wild-type FW213 and its derivatives in TH broth was monitored using a Bioscreen C reader (Oy Growth Curve AB Ltd.), as previously described (51). Briefly, bacterial cultures at an OD_600_ of 0.3 were diluted 1:50 in fresh TH broth and inoculated in the wells of a 96-well microtiter plate. All wells were overlaid with 50 μl sterilized mineral oil for anaerobic growth. Six biological repeats were used for each strain to estimate the generation time. The growth curves were constructed using GraphPad Prism 5.

### ECM binding assay.

The binding of *S. parasanguinis* strains to fibronectin, laminin, type I human collagen, or type IV human collagen was analyzed by an ELISA. The 96-well plates (enzyme immunoassay [EIA]/radioimmunoassay [RIA] plate; Costar) were coated with various amounts of ECM proteins as previously described (52). The wells were blocked with 200 μl of phosphate-buffered saline (PBS) containing 2% bovine serum albumin (BSA) at room temperature for 1 h prior to the addition of bacterial suspensions. Log-phase cultures (OD_600_ = 0.4) were washed once with PBS and resuspended in PBS to an OD_600_ of 0.4. An aliquot of 75 μl of the bacterial suspension was added to ECM molecule-coated wells and incubated at 37°C with 10% CO_2_ for 90 min. Wells with PBS only were used as a background control. At the end of the incubation, the wells were washed three times with 200 μl PBST (PBS with 0.1% Tween 20), followed by the addition of a monoclonal antibody specific for Fap1 (E42) of *S. parasanguinis* FW213 (53). Plates were incubated at room temperature for 1 h. Unbound E42 was removed by three washes with 200 μl PBST each. Horseradish peroxidase-conjugated goat anti-mouse IgG was then used to detect bound E42. The color reaction was developed using the ECL system (BD Biosciences). The reactions were stopped by the addition of 2 N H_2_SO_4_, and the final absorbance at 450 nm was recorded in a microplate reader (SoftMax Pro; Molecular Devices). Each assay was performed with triplicate samples.

### Examination of binding of *S. parasanguinis* FW213 to swine heart valves.

Swine hearts were purchased from a local wet market. Intact mitral valves were dissected from swine heart and then trypsin treated for 2 h as described previously (54). Intact swine heart valves were used as a negative control. Cultures of *S. parasanguinis* strains grown to an OD_600_ of 0.6 were washed once with PBS and resuspended at 1 × 10^10^ CFU ml^−1^ in PBS. A piece of trypsin-treated tissue (approximately 1 cm^2^) was placed in a 15-ml conical tube to which 1 ml of the bacterial suspension was added, and adherence was carried out for 90 min at 37°C in 10% CO_2_. The infected tissues were washed gently with PBS three times and then fixed in 10% formalin overnight. Goat anti-type I collagen affinity-purified polyclonal antibody (Millipore) was used to label exposed type I collagen, and mouse anti-lipoteichoic acid antibody (Novus) was used to localize *S. parasanguinis*. The locations of these primary antibodies were recognized by rhodamine-conjugated mouse anti-goat IgG and fluorescein isothiocyanate (FITC)-conjugated rabbit anti-mouse IgG, respectively. Samples were examined using confocal laser scanning microscopy (CLSM) (LSM510 Meta microscopy; Zeiss) with a 63× oil immersion lens objective. The images were displayed with LSM Image Browser software.

### Preparation of static and flow cell biofilms for CLSM examination.

To examine the biofilm structure by CLSM, static biofilms were prepared using an 8-well Nunc chamber slide system (Thermo Scientific). Briefly, early-log-phase cultures (OD_600_ = 0.3) were diluted 1:10, and 400 μl of the diluted cultures was inoculated into the wells of an 8-well chamber. The biofilm was grown for 24 h at 37°C. Biofilm cultures in the flow cell system were prepared as previously described (51), with minor modifications. Briefly, early-log-phase cultures were adjusted to an OD_600_ of 0.1 in fresh BMG. A 250-μl aliquot of the diluted cultures was injected into the growth chamber. The chamber was left upside down without medium flow for 4 h at 37°C. The medium pump was set at 5 ml^−1^ h^−1^ per channel, and the chamber was incubated for 18 h at 37°C.

Both the static and flow cell biofilms were stained with SYTO-9 and propidium iodide (PI) (Live/Dead biofilm viability kit; Invitrogen) according to the manufacturer’s recommendations. The stained biofilms were examined by CLSM with a 63× oil immersion lens objective. The biofilm images were displayed using Imaris 7.2.3 (Imaris).

### Quantitation of live/dead cells in static biofilms by flow cytometry.

Static biofilm cultures were prepared in 24-well plates, stained with SYTO-9 and PI as described above, and then analyzed by flow cytometry (55) to quantify the percentage of dead cells. For each sample, bacterial cells from three wells were used. An equal volume of 1× PBS was added to the stained bacterial suspension prior to flow cytometry analysis using a FACSCalibur instrument (BD Biosciences). The data were analyzed using CELLQuest software (BD Biosciences).

### Virulence of *S. parasanguinis* FW213 in the G. mellonella model.

G. mellonella larvae with a body weight of approximately 220 ± 50 mg were used in this study. Cultures of *S. parasanguinis* strains grown overnight in TH broth were washed twice with PBS and resuspended in PBS at 1.0 × 10^9^ CFU ml^−1^. Ten microliters of the bacterial suspension was injected into the proleg of each larva with a 26-gauge needle (Hamilton). PBS alone and heat-inactivated (75°C for 30 min) bacterial suspensions were used as negative controls. The infected larvae were kept at 37°C in a petri dish, without feeding for 14 days, and survival was monitored daily until the death of the negative controls. Larvae were scored as dead when they displayed no movement in response to touch. Twenty larvae were infected with each strain. Survival curves were constructed using the Kaplan-Meier method, and all data were analyzed by a log rank test using GraphPad Prism 5.

### Determination of the phagocytosis efficiency in Raw264.7 macrophages by flow cytometry.

To determine the impact of CBPs on the phagocytosis efficiency in Raw264.7 macrophages, early-log-phase cultures (OD_600_ = 0.3) of *S. parasanguinis* cells were labeled with FITC (isomer I). Briefly, bacteria were washed twice with PBS and then resuspended in FITC (0.1 mg ml^−1^) at approximately 5 × 10^9^ CFU ml^−1^. Labeling was carried out at room temperature for 1 h. At the end of the incubation, bacteria were washed twice with PBS prior to use. Mouse Raw264.7 macrophages were seeded in 6-well plates at 1.2 × 10^6^ cells per well in Dulbecco’s modified Eagle’s medium (DMEM) containing 10% fetal bovine serum (FBS) and 2 mM l-glutamine and allowed to adhere to plastic plates for 12 h prior to infection with bacteria. The macrophages were infected with labeled bacteria in DMEM without 10% FBS at MOIs of 10 and 50 for 15 min at 37°C in a 10% CO_2_ atmosphere. At the end of the infection, the extracellular bacteria were removed by three washes with PBS. The cell monolayers were harvested and then fixed with 2% paraformaldehyde in PBS at 4°C for 15 min. Cells were washed once with PBS, resuspended in 1% formaldehyde, and kept at 4°C overnight. An equal volume of PBS was added to each sample before analyzing the cell suspension using FACSCalibur. The data were analyzed using CELLQuest software.

### Macrophage survival assay.

Mouse Raw264.7 macrophages were seeded in 6-well plates at 1.2 × 10^6^ cells per well in DMEM containing 10% FBS and 2 mM l-glutamine and allowed to adhere to plastic plates for 12 h prior to infection with *S. parasanguinis*. All bacterial strains were grown to an OD_600_ of 0.3, washed twice with PBS, and resuspended in DMEM at approximately 5 × 10^9^ CFU ml^−1^. Raw264.7 macrophages were infected with bacteria at a MOIs of 10 and 50 for 30 min. At the end of the infection, the cell monolayer was washed with PBS twice, and the remaining extracellular bacteria were killed by the addition of penicillin at 100 U ml^−1^, streptomycin at 100 μg ml^−1^, and gentamicin at 100 μg ml^−1^ at 37°C for 1 h. The macrophage monolayer was washed with PBS, harvested, and lysed in PBS containing 0.1% Triton X-100 for 10 min. The intracellular viable bacterial counts were determined by serial dilution and plating.

### Statistical analysis.

Significant differences between wild-type FW213 and its derivatives were analyzed using one-way analysis of variance (ANOVA) followed by Dunnett’s test or Tukey’s test using GraphPad Prism 5. Differences were considered significant if the *P* value was <0.01.
