# Supplementary figures and images for: Functional Analysis of the Collagen Binding Proteins of Streptococcus parasanguinis FW213
Source: mSphere. 2020 Oct 14;5(5):e00863-20. doi: 10.1128/mSphere.00863-20 (PMC7565896; doi:10.1128/mSphere.00863-20)

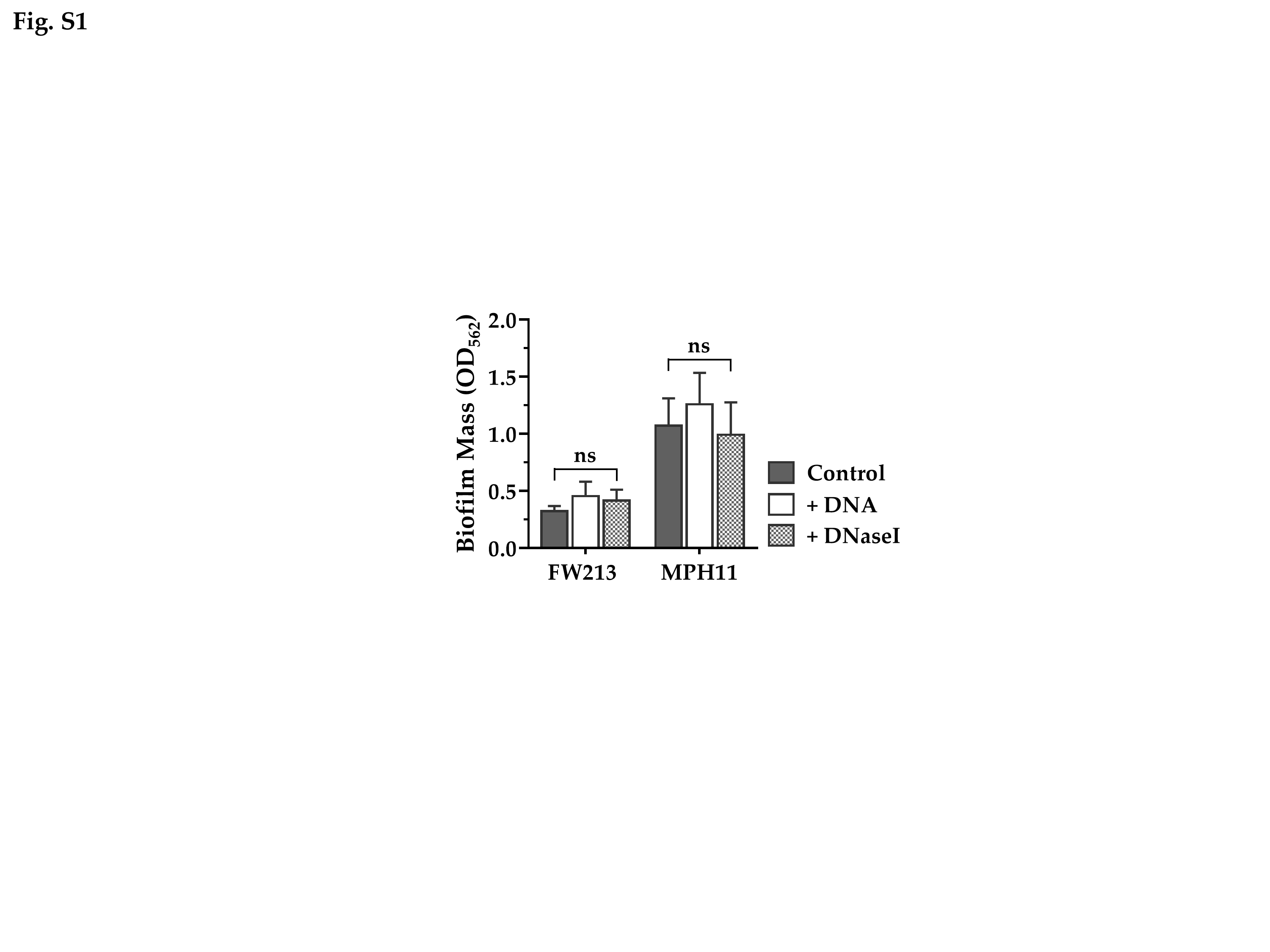

Supplement: FIG S1 [file mSphere.00863-20-sf001.tif]

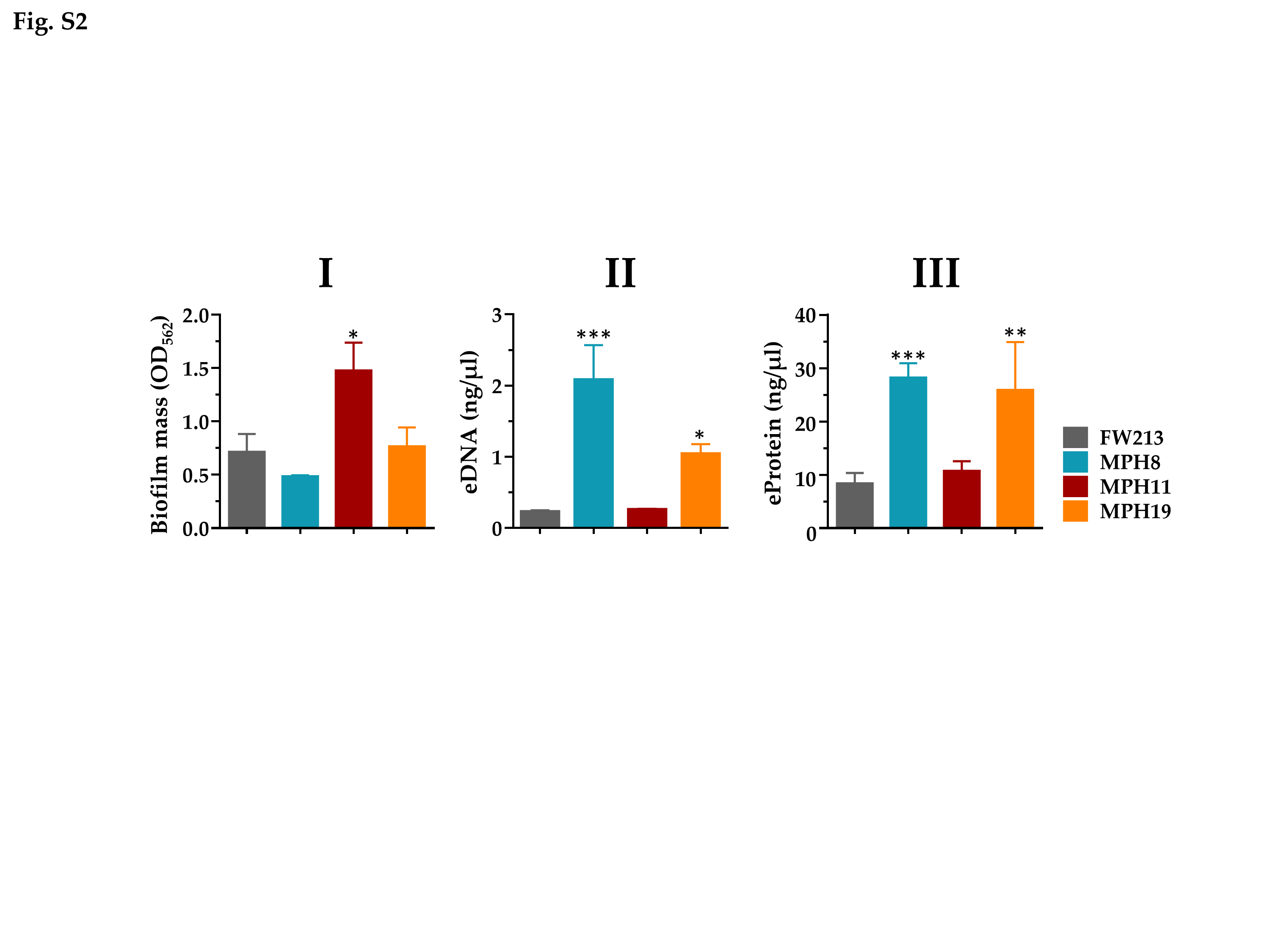

Supplement: FIG S2 [file mSphere.00863-20-sf002.tif]

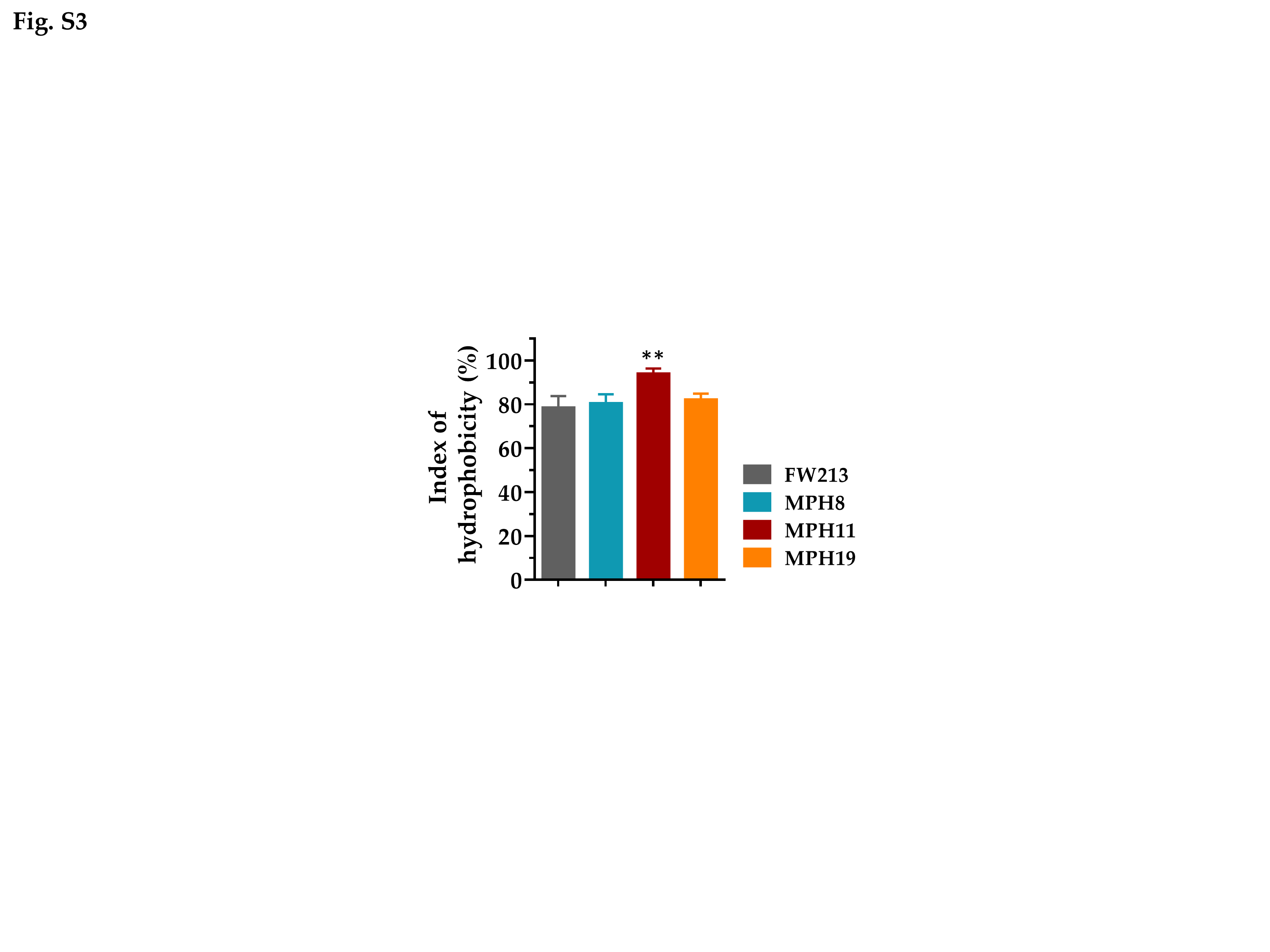

Supplement: FIG S3 [file mSphere.00863-20-sf003.tif]
